# Clinical profile, complications and outcome of scrub typhus in children: A hospital based observational study in central Nepal

**DOI:** 10.1371/journal.pone.0220905

**Published:** 2019-08-13

**Authors:** Santosh Pathak, Nagendra Chaudhary, Prativa Dhakal, Disuja Shakya, Prativa Dhungel, Gagan Neupane, Sandeep Shrestha, Shanti Regmi, Om P. Kurmi

**Affiliations:** 1 Department of Pediatrics, Chitwan Medical College, Bharatpur, Nepal; 2 Department of Pediatrics, Universal College of Medical Sciences, Bhairahawa, Nepal; 3 Department of Woman's Health and Development, School of Nursing, Chitwan Medical College, Bharatpur, Nepal; 4 Shreegaun Primary Health Care Centre, Dang, Nepal; 5 Population Health Research Institute, Department of Medicine, McMaster University, Hamilton, Canada; 6 Division of Respirology, Department of Medicine, McMaster University, Hamilton, Canada; University of Minnesota, UNITED STATES

## Abstract

**Background:**

Scrub typhus, an important cause of unexplained fever, is grossly neglected and often misdiagnosed in low and middle income countries like Nepal. The main aim of this study was to report on the clinical profile and complications of scrub typhus and its outcome in Nepalese children.

**Methods:**

A prospective observational study was carried out in children aged 1–16 years, admitted to a tertiary care hospital of central Nepal in between July 2016- Aug 2017. Scrub typhus was diagnosed with IgM ELISA.

**Results:**

All cases of scrub typhus (n = 76) presented with fever and commonly had other symptoms such as headache (75%), myalgia (68.4%), vomiting (64.5%), nausea (59.2%), abdominal pain (57.9%), cough (35.5%), shortness of breath (22.4%), altered sensorium (14.5%), rashes (13.2%) and seizures (11.8%). Important clinical signs noticed were lymphadenopathy (60.5%), hepatomegaly (47.4%), edema (26.3%), jaundice (26.3%), and splenomegaly (15.8%). About 12% (n = 9) had necrotic eschar. Similarly, thrombocytopenia, raised liver enzymes and raised creatinine values were seen in 36.9%, 34.2% and 65.8% respectively. The most common complications were myocarditis (72.4%), hypoalbuminemia (71.1%), severe thrombocytopenia (22.4%), renal impairment (65.8%), hyponatremia (48.7%) and hepatitis (34.2%). Over two-thirds (69.70%) of the cases were treated with doxycycline followed by combination with azithromycin in the remaining 18.4%. Overall, mortality rate in this group was 3.9%.

**Conclusions:**

Scrub typhus should be considered as a differential in any community acquired acute undifferentiated febrile illness regardless of the presence of an eschar. Myocarditis and acute kidney injury are important complications which when addressed early can prevent mortality. Use of doxycycline showed a favorable outcome.

## Introduction

Scrub typhus, an important cause of tropical fever in northern Australia, the western Pacific islands and South Asia including India and Nepal, is caused by a zoonotic obligate intracellular, gram negative bacteria, transmitted by a bite of trombuculid mite, the chigger [1, 2].The disease is grossly under-diagnosed in low and middle income countries (LMICs) as the presentation and index of suspicion is low among clinicians. Limited awareness about the disease and lack of diagnostic facilities in developing nations like Nepal are also important reasons for the under-diagnosis of the disease and delay in specific treatment have been reported to be associated with increased case fatality rates [2].

Scrub typhus in Nepal was first ever reported in 1981 and a hospital based study carried out in 2004 found 28 cases of scrub typhus among 876 enrolled febrile patients [3]. In a recent study (2017) conducted in National Public health laboratory (Nepal) reported that 40.3% of blood samples collected from patients with acute febrile illness were positive for IgM against *O*. *tsutsugamushi* [4].

Scrub typhus can manifest with nonspecific symptoms, such as fever, myalgia, hepatosplenomegaly, lymphadenopathy and thrombocytopenia and hence it should be considered as an important cause of acute undifferentiated fever in tropical and subtropical regions [5–7].

Although few studies have been reported previously, the data of scrub typhus in children is scarce. Therefore, a hospital based study was conducted in Chitwan district of central Nepal to study the clinico-laboratory profile and therapeutic outcome of scrub typhus in children aged 1–16 years of life.

## Methods

### Ethical approval

The study was approved by the Institute research Committee (IRC) of Chitwan Medical College, Bharatpur, Nepal *(Reference number-CMC-IRC-112*, *dated 30 June*, *2016)*. Written and oral consents were taken from the informants.

### Hospital setting, patient selection and diagnosis

A prospective observational study was conducted at Chitwan Medical College Teaching Hospital (CMC-TH), a tertiary care referral teaching hospital situated in central region of Nepal over a period of 14 months (1^st^ July 2016- 31^st^ Aug 2017). Children aged 1–16 years were included in the study. All suspected cases with fever (n = 312) but without any identifiable infection along with presence of one or more of the following clinical features (rashes, edema, hepatosplenomegaly, lymphadenopathy and eschar) were subjected to serological test for scrub typhus. Common infectious conditions such as that could clinically mimic scrub typhus were ruled out by performing the following tests: peripheral smear and rapid antigen test for malaria, dengue (NS1 antigen and IgM antibody) test, urine and blood cultures as per clinical aspect. Leptospira serology and an HIV-ELISA were performed when clinically indicated. Cardiac evaluation (Echocardiography and CPK-MB) and cerebrospinal fluid (CSF) analysis was performed for selected cases with suspected myocarditis or meningoencephalitis respectively. Serological diagnosis for scrub typhus was made by IgM ELISA test (In BiOS International, Inc. Seattle USA). A favorable clinical response with oral doxycycline and azithromycin (defervescence within 48 hours) was considered an additional evidence of the disease. Diagnosed cases (n = 76) were treated with a 7–10 day course of antibiotics [doxycycline 5–7 mg/kg/day twice daily and few were treated with doxycycline plus ceftriaxone (considering other co-infection) while those showing poor response with doxycycline (fever persisting even after 72 hours of therapy) and ceftriaxone were treated with oral or intravenous azithromycin for 5 days]. Children who did not tolerate doxycycline and had nausea and vomiting soon after its intake were given intravenous azithromycin instead of injectable doxycycline, as injectable doxycycline is not available in Nepal. Chloramphenicol or rifampicin was not used in our center for any patients. The response to treatment, the defervescence time, and the complications were noted. Children included in the study were followed up to discharge, mortality or leave against medical advice (LAMA).

### Definitions

The following criteria were used to define the various complications in scrub typhus.

■ Acute Kidney Injury (AKI): Rise of serum creatinine of at least 0.3 mg/dl or 50% higher than baseline within a 24–48 hour period or a reduction in urine output to 0.5 mL/kg per hour for longer than 6 hours.
■ Acute hepatitis: Elevation of serum transaminases more than 2 times the normal upper limit.
■ Meningoencephalitis: Altered sensorium along with signs of meningeal irritation and/or seizures associated with elevated protein and lymphocytic/neutrophilic cytology with normal or low sugar on CSF analysis.
■ Multiple Organ Dysfunction Syndrome (MODS): Dysfunction of more than one organ, requiring intervention to maintain homeostasis.
■ Myocarditis: Child with tachypnea, tachycardia and/or (S_3_- gallop, shock) along with echocardiographic finding suggestive of reduced ejection fraction and elevated cardiac enzymes (CPK-MB).
■ Hyponatremia: Serum sodium level less than 135 meq/L.
■ Hypoalbuminemia: Serum albumin level less than 2.5 gm/dl.

### Variables

Clinical data, including the duration of fever, associated symptoms, vital signs, and the general and systemic examination findings, were recorded. A careful search for eschar was performed in all patients. Data regarding age, sex and residential area were collected. Complete blood counts, chest X-rays, renal function test (urea, creatinine), liver function test (serum bilirubin, aspartate transaminase, alanine transaminase, serum albumin, total protein and alkaline phosphatase)) and urinalysis were performed at the time of presentation for all cases and were repeated if necessary.

### Statistical analysis

The data were analyzed using SPSS software, version 16.0 (SPSS Inc., Chicago, IL). Descriptive statistics in terms of frequency, percentage, mean and standard deviation were calculated.

## Results

### Clinico-demographic profile of scrub typhus cases

Out of 312 cases tested for scrub typhus serology, 24.4% (n = 76) were diagnosed with scrub typhus with age ranging from 2 to 16 years with mean age of 8.8±3.8 years and 64.5% (n = 49) being male. Most (83.3%) of the children were diagnosed in between the months of September and November. More than one third (42.11%) of the children were from Chitwan district, the district where the tertiary hospital was located, 28.9% from the neighboring district of Nawalparasi (28.95%) and the remaining 9.2% from other districts (Lamjung, Parbat, Rolpa, Bara, Dang and Baglung).

Table 1 shows the clinico-demographic profile of children at the time of admission.

**Table 1:** Clinico-demographic profile of scrub typhus.

Clinical manifestations	N	%	Demographic data	N	%
**Fever**	76	100	**Age**		
***<5 days***	33	43.4	*2–5 years*	19	25
***>5 days***	43	56.6	*6–10 years*	26	34.2
**Headache**	57	75	*11–16 years*	31	40.8
**Myalgia**	52	68.4	**Sex**		
**Vomiting**	49	64.5	*Male*	49	64.5
**Lymphadenopathy**	46	60.5	*Female*	27	35.5
**Nausea**	45	59.2	**Habitat**		
**Pain abdomen**	44	57.9	*Chitwan*	32	42.1
**Hepatomegaly**	36	47.4	*Nawalparasi*	22	28.9
**Dry cough**	27	35.5	*Makwanpur*	8	10.5
**Edema**	20	26.3	*Gorkha*	7	9.2
**Jaundice**	20	26.3	*Others*	7	9.2
**Shortness of breath**	17	22.4			
**Splenomegaly**	12	15.8			
**Altered sensorium**	11	14.5			
**Rashes (maculopapular)**	10	13.2			
**Seizures (GTCS)**	9	11.8			
**Eschar**	9	11.8			

All children (n = 76) presented with fever. Among them, (43.4% (n = 33) had history of fever for less than 5 days and remaining 56.6% (n = 43), for more than 5 days. High grade fever (>101°F) was recorded in 85.5% (n = 65) children during admission. Other common symptoms were headache, myalgia, vomiting, nausea, abdominal pain, dry cough, shortness of breath, altered sensorium, maculopapular rashes and generalised tonic clonic seizures (GTCS) respectively. Important clinical signs noticed on examination included lymphadenopathy (60.5%), hepatomegaly (47.4%), edema (26.3%), jaundice (26.3%), splenomegaly (15.8%). A necrotic eschar which is considered as most useful diagnostic sign for scrub typhus was present in 11.8% (n = 9) children. Groin and axilla was the most common site for eschar seen in our study.

### Laboratory parameters of children with scrub typhus

Tables 2 and 3 depict the laboratory parameters of the children with scrub typhus.

**Table 2:** Laboratory values of children with scrub typhus.

Characteristics	Mean ± SD
**Total leucocyte count (cells/mm^3^)**	9053.73 ± 6222.25
**Platelet count (cells/mm^3^)**	141381.6±115976.4
**Bilirubin (mg/dl)**	
***Total***	2.7 ± 2.8
***Direct***	1.1 ± 1.7
**Aspartate transaminase (AST) (IU/L)**	343.3 ± 864.2
**Alanine transaminase (ALT) (IU/L)**	291.9 ± 808.7
**Serum albumin (gm/dl)**	3 ± 0.7
**Prothrombin time (PT) (secs)**	15.1 ± 4.2
**International normalised ratio (INR)**	1.2 ± 0.3
**Urea (mg/dl)**	46.2 ± 41.7
**Creatinine (mg/dl)**	0.8 ± 0.5

AST, aspartate transaminase; ALT, alanine transaminase; PT, prothrombin time; INR, International normalised ratio.

**Table 3:** Laboratory findings of children with scrub typhus.

**Biochemical parameters**		
	N	%
**Raised bilirubin**		
**Direct (>2 mg/dl)**	15	19.7
**Indirect (>1.2 mg/dl)**	46	60.5
**Hypoalbuminemia (<2.5 gm/dl)**	54	71.1
**Hyponatremia (<135 meq/L)**	37	48.7
**Raised liver enzymes (AST and ALT >45 IU/L)**	26	34.2
**Raised creatinine (>1.5 mg/dl)**	50	65.8
**Hematological Parameters**		
**Platelet count (cells/mm^3^)**		
***10000–50000***	17	22.4
***50001–100000***	11	14.5
***100001–150000***	22	28.9
***150001–333000***	26	34.2

Out of 76 cases, 11 (14.5%) had platelet count between 50001 to 10000/mm^3^ and 17 (22.4%) had between 10000 to 50000/mm^3^. Fifteen (19.7%) had direct bilirubin >2mg/dl and 46(60.5%) had indirect bilirubin >1.2 mg/dl. Similarly, 22 (28.9%) had serum albumin >3.5gm/dl and 37 (48.7%) had sodium level below 135 meq/l. Raised liver enzymes and raised creatinine values were seen in 34.2% and 65.8% respectively.

### Complications of scrub typhus

Table 4 shows the most common complications were myocarditis (n = 55, 72.4%), hypoalbuminemia (n = 54, 71.1%), renal impairment (n = 50, 65.8%), hyponatremia (n = 37, 48.7%), hepatitis (n = 26, 34.2%) and severe thrombocytopenia (22.4%). Among 76 cases, 42 were managed in high dependency unit (HDU), 20 in general ward and 14 cases required pediatric intensive care unit (PICU) admission. Majority of children with myocarditis (S_3_-gallop) received dobutamine (+/- low dose of diuretics) and those with shock received fluid boluses along with inotropes. Inotropes were given to 14 cases. Children with catecholamine resistant shock, worsening respiratory distress and Glasgow Coma Scale (GCS) less than 7 required ventilatory support. Out of 8 children received who ventilatory support, three of them expired due to multi-organ dysfunction syndrome. None of the children with AKI needed dialysis. Platelet transfusion was considered in all children with platelet count less than <20,000/mm^3^. Children with meningoencephalitis who showed increased CSF cell count (predominantly lymphocytic, few neutrophilic), raised protein and low sugar were treated as per protocol.

**Table 4:** Complications of scrub typhus.

Characteristics	Frequency (N)	Percentage (%)
**Cardiac Dysfunction (myocarditis)**	55	72.4
**Hypoalbuminemia**	54	71.1
**Severe thrombocytopenia (<50,000/mm^3^)**	17	22.4
**Acute kidney injury**	50	65.8
**Hyponatremia**	37	48.7
**Hepatitis**	26	34.2
**Multi organ dysfunction syndrome (MODS)**	3	3.9

Doxycycline was commonly used (69.7%) in the treatment of scrub typhus followed by combination with azithromycin (18.4%). 67 (88.2%) recovered well and were discharged, 3 (3.9%) died due to multi-organ failure and 6 (7.9%) were discharged due to other reasons with lost to follow up (Table 5).

**Table 5:** Treatment and clinical outcomes of children with scrub typhus.

Treatment	Frequency	Percentage
**Antibiotics Used**		
***Doxycycline only***	53	69.7
***Ceftriaxone and Azithromycin***	3	3.9
***Doxycycline and Azithromycin***	14	18.4
***Others***	6	7.9
**Clinical Outcomes**		
***Recovered and discharged***	67	88.2
***Death***	3	3.9
***Referred***, ***LAMA***	6	7.9

## Discussion

The data of clinico-laboratory profile as well as the outcome of scrub typhus in children in Nepal is scarce. Males were more likely to be affected compared to females (64.5% versus 35.5%) [8–10]. The mean age of children in the present study was 8.8 years with three-quarter in between 6–16 years similar to findings reported by Bhat et al (2014) from north India [7]. The incidence of scrub typhus increased as the age increased in the present study which could be due to frequent exposures of chiggers in older children especially males while playing outdoor.

The clinical manifestations in scrub typhus are nonspecific and have wide variations in presentation. Majority of children in the present study were symptomatic (with fever, headache and myalgia). Recent studies conducted by Kumar et al and Palanivel et al also demonstrated fever to be present in all cases [7, 11].Hepatomegaly was seen in in 47.4% whereas edema and jaundice were noticed in 26.3% each. Edema has been reported from 37% up to 60% in two studies from India [7, 11]. Lymphadenopathy in the present study was 60.5% which was almost similar to a study done in Srilanka by Silva et al [12] while Kumar et al found low incidence of lymphadenopathy in their study [13]. Eschar in the present study was 11.8%, similar to earlier studies from India [6, 14]. A recent study (2019) in India by Bal et al in 201 children with scrub typhus demonstrated the presence of eschar in 17.9% cases [15].

However, few studies have reported the presence of eschar in 50–80% cases of scrub typhus [8, 16, 17]. In contrary, some authors have reported scrub typhus cases even without the presence of eschar. Presence of eschar in a child with fever, thrombocytopenia and renal impairment can be a useful clinical tool in excluding dengue infection in children with scrub typhus [2, 10, 18, 19].

Previous studies have observed rashes ranging from 15–91% but in our study, rashes were observed only in 13.2% of cases [8, 16–18, 20].

Myocarditis (72.4%), hypoalbuminemia (71.1%) and AKI (65.8%) were three major complications seen in children with scrub typhus in the present study. Majority of children with myocarditis had shock at presentation and required fluid, diuretic therapy, fluid restriction, boluses and inotropic support as required. None of the AKI cases required dialysis as they improved with conservative management. The reason for renal impairment in majority of cases with scrub typhus may be due to multi-organ involvement/failure whereas pre-renal cause due to intravascular fluid depletion could also be another reason for acute kidney injury. The comparison of complications in the present study with other previous studies have been depicted in Table 6. Although the proportion of complications in a study done by Kumar et al. was low than the present study, myocarditis (34%), hypoalbuminemia (54%) and AKI (20%) comprised three leading complications in their study [6]. Another recent longitudinal study (2016) in Meghalaya (India) in 90 scrub typhus cases with mean age (SD) 36.3 (13.4) years showed that acute hepatitis (n = 15, 16.7%), pneumonitis (n = 14, 15.6%) and AKI (n = 11, 12.2%) were common complications with 38.5% (n = 5) death due to MODS. This suggests that the complication rate in children are much higher when compared to adults with scrub typhus [21].

**Table 6:** Comparison of complications in the present study with other previous studies.

Complications	Present Study	Kumar et al [6]	Chanta et al [16]	Hung et al [8]	Somashekhar et al [14]
**Myocarditis**	72.4	34			
**Hypoalbuminemia**	71.1	54			
**Thrombocytopenia (<100,000/mm^3^)**	36.9	32	80	50	63.4
**Acute kidney injury (AKI)**	65.8	20			
**Hyponatremia**	48.7	17			
**Hepatitis**	34.2	31	75	91.3	70.7
**Meningoencephalitis**	14.5	17	5	17.9	14.6

A recent Cochrane review (2018) concluded that tetracycline, doxycycline, azithromycin, and rifampicin are effective drugs available for the treatment of scrub typhus and suggested that there may be little or no difference between tetracycline, doxycycline, and azithromycin as treatment options. The review also found that there were few treatment failures with the above mentioned drugs [22]. Majority of children in the present study were treated with doxycycline and showed a favorable clinical response. Previous studies have also shown a similar clinical response to doxycycline [8, 16, 18, 23]. Mortality rate in our study (3.9%) was lower than the study conducted by Kamarasu et al (15%) and Rathi et al (9%) [10, 24].

The present study has some limitations. As the study was conducted in a tertiary care referral hospital, the present findings may not reflect the exact burden of the disease in the community. Scrub typhus was diagnosed with IgM ELISA as indirect immunofluorescence test which is considered as the gold standard diagnostic test and is not easily available in many hospitals in Nepal.

## Conclusion

Scrub typhus should be considered as a differential in any community acquired undifferentiated febrile illness regardless of the presence of an eschar, and needs empirical therapy along with testing for scrub typhus. Myocarditis and acute kidney injury are important complications which when addressed early can prevent mortality. Use of doxycycline shows a favorable outcome.

## Supporting information

S1 Checklist STROBE checklist. (DOC)

S1 Dataset Database. Variables data of scrub typhus.
(XLSX)
