# Supplementary material for: Clinical profile, complications and outcome of scrub typhus in children: A hospital based observational study in central Nepal
Source: PLoS One. 2019 Aug 13;14(8):e0220905. doi: 10.1371/journal.pone.0220905 (PMC6692021; doi:10.1371/journal.pone.0220905)
Supplement: S1 Checklist — (DOC) [file pone.0220905.s001.doc]

STROBE Statement—Checklist of items that should be included in reports of ***cross-sectional studies***

|  | Item No | Recommendation |
| --- | --- | --- |
| **Title and abstract** | 1 | (*a*) Indicate the study’s design with a commonly used term in the title or the abstract  **Study design mentioned**   - **In the title, Page 1, line 2** - **In abstract section: page 2, line 28** |
|  |  | (*b*) Provide in the abstract an informative and balanced summary of what was done and what was found.  **Line 32-45 (page 2)**  - **Clinical profile, complications and outcome was studies in all children and the findings have been mentioned in the abstract.** |
| Introduction | | |
| Background/rationale | 2 | Explain the scientific background and rationale for the investigation being reported  ***In the introduction section; Line 57-78 (page 3-4***):  **Scrub typhus is grossly under-diagnosed in low and middle income countries (LMICs) as the presentation and index of suspicion is low among clinicians. Limited awareness about the disease and lack of diagnostic facilities in developing nations like Nepal are also important reasons for the under-diagnosis of the disease and delay in specific treatment have been reported to be associated with increased case fatality rates.** |
| Objectives | 3 | State specific objectives, including any prespecified hypotheses  **Line 76-78 (Page 5):**  **Therefore, a hospital based study was conducted in Chitwan district of central Nepal to study the clinico-laboratory profile and therapeutic outcome of scrub typhus in children aged 1-16 years of life.** |
| Methods | | |
| Study design | 4 | Present key elements of study design early in the paper  **Page 5 (line 87-88)**  **An prospective observational study was conducted at Chitwan Medical College Teaching Hospital (CMC-TH), a tertiary care referral teaching hospital situated in central region of Nepal over a period of 14 months (1^st^ July 2016- 31^st^ Aug 2017).** |
| Setting | 5 | Describe the setting, locations, and relevant dates, including periods of recruitment, exposure, follow-up, and data collection  **Line 83-104 (page 4 and 5)**  **Setting, location, time of recruitment, cases enrolled, variables studied with complications and outcome have been mentioned.** |
| Participants | 6 | 1. Give the eligibility criteria, and the sources and methods of selection of participants   **Page 4 (line 85-88)**  **All suspected cases with fever but without any identifiable infection along with presence of one or more of the following clinical features (rashes, edema, hepatosplenomegaly, lymphadenopathy and eschar) were included in the study. Serological diagnosis was made by IgM ELISA test (In BiOS International, Inc. Seattle USA).** |
| Variables | 7 | Clearly define all outcomes, exposures, predictors, potential confounders, and effect modifiers. Give diagnostic criteria, if applicable  **Given in Method section (Pages 5-8), lines 81-146** |
| Data sources/ measurement | 8* | For each variable of interest, give sources of data and details of methods of assessment (measurement). Describe comparability of assessment methods if there is more than one group  **Given in the Method section (pages 5-8, lines 81-146)** |
| Bias | 9 | Describe any efforts to address potential sources of bias  **All febrile suspected cases were tested for scrub typhus serology (given in the method section)** |
| Study size | 10 | Explain how the study size was arrived at  **All febrile cases were recruited during the study period (given in the method section)** |
| Quantitative variables | 11 | Explain how quantitative variables were handled in the analyses. If applicable, describe which groupings were chosen and why  **All variables were recorded in a excel sheet and mean and percentages were calculated for each variables in scrub typhus cases.** |
| Statistical methods | 12 | 1. Describe all statistical methods, including those used to control for confounding   **Mean and percentages were used.** |
|  |  | 1. Describe any methods used to examine subgroups and interactions   **None** |
|  |  | 1. Explain how missing data were addressed   **There were no missing data** |
|  |  | 1. If applicable, describe analytical methods taking account of sampling strategy   **None** |
|  |  | 1. Describe any sensitivity analyses   **None** |
| Results | | |
| Participants | 13* | 1. Report numbers of individuals at each stage of study—eg numbers potentially eligible, examined for eligibility, confirmed eligible, included in the study, completing follow-up, and analysed   **Page 9 (lines 149-151) in Results section:**  **Out of 312 cases tested for scrub typhus serology, 24.4% (n=76) were diagnosed with scrub typhus with age ranging from 2 to 16 years with mean age of 8.8±3.8 years and 64.5%(n=64) being male.** |
|  |  | 1. Give reasons for non-participation at each stage   **Mentioned in the result section** |
|  |  | 1. Consider use of a flow diagram   **Mentioned in the result section which is clear (flow diagram not necessary)** |
| Descriptive data | 14* | 1. Give characteristics of study participants (eg demographic, clinical, social) and information on exposures and potential confounders   **Mentioned in the result section** |
|  |  | 1. Indicate number of participants with missing data for each variable of interest   **Mentioned in result section** |
| Outcome data | 15* | Report numbers of outcome events or summary measures  **Lines 148-214, pages 8-14** |
| Main results | 16 | 1. Give unadjusted estimates and, if applicable, confounder-adjusted estimates and their precision (eg, 95% confidence interval). Make clear which confounders were adjusted for and why they were included   **See result section, for main results** |
|  |  | (*b*) Report category boundaries when continuous variables were categorized |
|  |  | 1. If relevant, consider translating estimates of relative risk into absolute risk for a meaningful time period   **Not applicable** |
| Other analyses | 17 | Report other analyses done—eg analyses of subgroups and interactions, and sensitivity analyses  **Not applicable** |
| Discussion | | |
| Key results | 18 | Summarise key results with reference to study objectives  **Pages 15-19, lines 219-283** |
| Limitations | 19 | Discuss limitations of the study, taking into account sources of potential bias or imprecision. Discuss both direction and magnitude of any potential bias  **Page 19, Lines 279-283** |
| Interpretation | 20 | Give a cautious overall interpretation of results considering objectives, limitations, multiplicity of analyses, results from similar studies, and other relevant evidence  **Given in discussion section** |
| Generalisability | 21 | Discuss the generalisability (external validity) of the study results |
| Other information | | |
| Funding | 22 | Give the source of funding and the role of the funders for the present study and, if applicable, for the original study on which the present article is based  **None** |

*Give information separately for exposed and unexposed groups.

**Note:** An Explanation and Elaboration article discusses each checklist item and gives methodological background and published examples of transparent reporting. The STROBE checklist is best used in conjunction with this article (freely available on the Web sites of PLoS Medicine at http://www.plosmedicine.org/, Annals of Internal Medicine at http://www.annals.org/, and Epidemiology at http://www.epidem.com/). Information on the STROBE Initiative is available at www.strobe-statement.org.
